# Chronic Illness, Subjective Wellbeing, and Health Services Availability: A Study of Older Adults in Australia

**DOI:** 10.3390/ijerph18157718

**Published:** 2021-07-21

**Authors:** Siqin Wang, Yan Liu, Jack Lam, Zhe Gao

**Affiliations:** 1 School of Earth and Environmental Sciences, The University of Queensland, St Lucia 4067, Australia; s.wang6@uq.edu.au (S.W.); yan.liu@uq.edu.au (Y.L.); 2 Institute for Social Science Research, The University of Queensland, Indooroopilly 4068, Australia; j.lam@uq.edu.au; 3 Hubei Provincial Key Laboratory for Geographical Process Analysis and Simulation, Central China Normal University, Wuhan 430079, China

**Keywords:** chronic illnesses, subjective wellbeing, older adults, health service availability, Australia

## Abstract

Chronic illness is prevalent in older adults. While current scholarship has examined how various factors may be associated with the onset of chronic illnesses, fewer scholars have examined the role of health services availability. Drawing on a sample of older adults aged 50 and above from wave 16 of the Household, Income, and Labour Dynamics in Australia survey and geo-coded information of general practitioners (GPs) from the Australian Medical Directory, 2016, we investigated whether living in areas with a greater number of GPs is related to reports of living with a chronic illness. Contrary to our hypothesis, we did not find an association between the availability of health services and reports of chronic illnesses, though factors such as better socioeconomic status and better subjective wellbeing are related to lower likelihoods of reporting a chronic illness. We concluded that, while easy access to local health services may be important for the diagnosis and treatment of chronic illnesses, it is less persuasive to attribute the availability of health services to the likelihood of older adults reporting chronic illnesses without knowing how much or how often they use the services.

## 1. Introduction

Chronic illness is common among older adults, with increasing prevalence as individuals age. According to the 2017–18 National Health Survey conducted by the Australian Bureau of Statistics (ABS), about one third of Australians at all ages and 78% of those aged 65 or over report at least one of nine types of chronic illnesses, including arthritis, asthma, coronary heart disease, chronic obstructive pulmonary disease, depression, type II diabetes, high blood pressure, osteoporosis, or cerebrovascular disease (stroke) [1]. Reducing the incidence of chronic illnesses―usually measured by the self-reported chronic illnesses―has become an important component of the health initiatives in many countries worldwide, with the advocacy of successful aging, longer life expectancy, and subjective wellbeing in later life [2].

An extensive body of research suggests that better mental wellbeing may protect against the onset of illnesses [3]. These studies have largely documented the link between negative emotions and subsequent health outcomes, including cardiovascular disease, disability, and mortality. It is evident that living with chronic illnesses is related to higher psychological distress [4,5,6,7]. However, such a relationship varies across individuals with different characteristics, such as gender [6], age [8], marital status [9], and socioeconomic status [10]. The way these individual characteristics are associated with the presence of chronic illnesses remains inconsistent, and is less often applied to the older adult population [11,12,13]. Furthermore, prior studies have given more attention to the individual-specific characteristics, but less on how factors such as the location of residence and the availability of health services may relate to the incidence of chronic illnesses. It is possible that older adults living in areas with better health services are less likely to develop chronic illnesses, as they are more readily able to check health conditions regularly and address any health issues prior to its onset.

Taking a sample of older adults aged 50 and above in Australia as the study population, this study aims to examine the associations between chronic illness, subjective wellbeing, and health service availability, taking into account the individual characteristics of the older adults. There are three research questions: (1) What are the differences in individual characteristics between older adults with and without chronic illnesses? (2) To what extent is subjective wellbeing related to the reporting of a chronic illness? (3) Is better health service availability associated with a lower likelihood of reporting chronic illness? Linking the geo-coded data of general practitioners from the Australian Medical Directory with survey data from wave 16 of the Household, Income, and Labour Dynamics in Australia (HILDA), we employed binomial logistic regressions to investigate the associations between subjective wellbeing, health service availability, and chronic illness. Our study contributes to building an ongoing evidence base that is particularly important for policy implications for successful aging and health service planning more broadly, and in Australia specifically.

## 2. The Construction of the Hypotheses

### 2.1. The Relationship between Chronic Illness and Individual Characteristics

Chronic illnesses are defined broadly as health conditions that last one year or longer and require ongoing medical attention or limit activities of daily living, or both [14]. An increasing number of incidences of chronic illnesses among the aging population has been observed [15], however, the role that their individual characteristics (i.e., demographic and socioeconomic status) may play in affecting the incidence of chronic illnesses remains less clear. Considering that our study used data on self-reported chronic illnesses collected from a nation-wide survey, we considered the expression “incidence of chronic illnesses” equivalent to “reporting chronic illnesses” in this study. Concerning the potential relationship between socioeconomic status and chronic illnesses, existing research shows that, in general, good socioeconomic positions are associated with better health. For instance, a systematic narrative review of studies conducted from 1995 to 2013 concerning older adults in Europe showed that older adults with lower income and a lower education level reported poorer health [16]. Furthermore, some studies also reported that people with better general health are associated with a lower likelihood of encountering chronic illnesses [11,17]. Linking the observed relationship between socioeconomic positions and general health and the reported relationship between general health and chronic illnesses, we may speculate that people in better socioeconomic positions may be associated with a lower likelihood of reporting chronic illnesses. More specifically, we hypothesise that older adults in better socioeconomic positions (e.g., well-educated and high income) might have better cognition and pay more attention to their physical health, and are therefore less likely to report chronic illness.

Furthermore, the relationship between socioeconomic status and chronic illnesses may also be affected by the individual’s demographic characteristics. For instance, it has been observed in different ethnic groups in South Africa that race, age, and gender are associated with the status of chronic illnesses, but this has not been investigated in other countries [11]. Studies conducted across different countries have indicated that age plays a key role in the increasing incidence of chronic illnesses [12,13,18,19,20]; however, other demographic features, such as gender and marital status, are rarely observed to be associated with chronic illnesses. Therefore, we drew our first set of hypotheses by taking into account the individual’s demographic features and socioeconomic positions based on the existing scholarship:

**Hypothesis** **1a** **(H1a).** *Older adults in better socioeconomic positions* *(e.g., higher income and well-educated) are less likely to report chronic illnesses;*

**Hypothesis** **1b** **(H1b).** *Older age is associated with a higher likelihood of reporting chronic illnesses;*

**Hypothesis** **1c** **(H1c).** *Gender and marital status are not associated with chronic illnesses.*

### 2.2. The Relationship between Chronic Illness and Subjective Wellbeing

Early studies focused more on the relationship between emotion and health conditions, especially negative emotion and subsequent health outcomes, including cardiovascular disease, disability, and mortality [4,5,6,7]. More recently, the relationship between psychological wellbeing and chronic illnesses of the general population has been studied [21]. The essential proposition is that wellbeing may provide a “broad base of resilience” to protect against the onset of chronic illnesses [20]. A higher level of subjective wellbeing is usually accompanied by positive emotions, such as hope, anticipation, joy, and happiness; such emotions play a protective role in the development of chronic illnesses, such as hypertension and diabetes mellitus [22]. However, the relationship between chronic illnesses and wellbeing may be subject to the types of chronic illnesses [3], as well as the measure and definition of wellbeing [23].

Western and Tomaszewski [24] define wellbeing with two components: subjective wellbeing and objective wellbeing. Subjective wellbeing can be measured in two distinct perspectives: hedonic wellbeing, which refers to the experience of positive emotion (e.g., pleasure, happiness, and life satisfaction), and eudaimonic wellbeing, which refers to longer-term functioning (e.g., self-realization or sense of autonomy) [24]. On the other hand, objective wellbeing can be measured by a series of indictors to reflect the objective components of a good life, including high income, material sufficiency, good relationships with family and friends, and positive life events [23,24]. Herein, this study extends from the definition by Western and Tomaszewski [24] to measure subjective wellbeing as the level of mental and physical health, integrating the dimensions of hedonic wellbeing, eudaimonic wellbeing, and objective wellbeing. More specifically, a high level of mental health can be reflected by both hedonic wellbeing as an indicator of positive emotion and eudaimonic wellbeing as an indicator of good self-realization [24]. A high level of physical health is a reflection of sound objective wellbeing as an indicator of healthy lifestyle, good living conditions, and sufficient living materials, nutrition, medicine, and health services [23,24,25]. These measures of subjective wellbeing in our study provide a more holistic capture of wellbeing for older adults.

Given the hypothesized effect of individual characteristics on chronic illnesses in Hypothesis 1, the relationship between subjective wellbeing and chronic illness may be further controlled by the individual characteristics of older adults [15,16,17,18,19,20]. More specifically, compared to older adults with financial burden and living pressure, the counterparts in better socioeconomic positions (e.g., higher income and well-educated) are more likely to have better living conditions and a sufficient provision of living materials [16,17,21], and are more able to sustain good mood and positive emotion towards their surroundings, preventing the incidence of chronic illnesses in the long-term [21,22]. Therefore, we formulated our second set of hypotheses as below:

**Hypothesis** **2** **(H2).** *Controlled by individual characteristics, older adults with better* *subjective wellbeing* *are less likely to report chronic illnesses.*

### 2.3. The Relationship between Chronic Illnesses and Health Service Availability

Unlike the common perception that proximity to health services is critical to emergent illnesses (e.g., heart disease, stroke, and broken bones), controversial views exist on whether the availability of health services at the local level influence the incidence of chronic illness [25]. From a psychological perspective, the availability of health services in local suburbs may matter for the incidence of chronic illness, as individuals may feel more comfortable knowing that health services are available nearby [26]. Moreover, a larger number of health services available locally may suggest more options for the older adults in managing their illness, and in obtaining the social and health support needed [27]. As a South African study shows, 71% of its study population were reliant on public health facilities for treatment, suggesting that people would experience considerable difficulty managing their chronic conditions if there were not health services near where they live [11]. This may be particularly important for older adults with less mobility for physical activity. Older adults with a chronic illness would be diagnosed earlier, receive healthcare more frequently, and prevent the occurrence/worsening of chronic conditions in long-term should they have easy access to local health services. In the Australian context, rural communities are subject to poorer health status and increased problems of accessing health services compared with their metropolitan counterparts [28]. Difficulties of accessing health services may result from the need to overcome distance barriers and the limited local availability of health services due to the high costs of providing such services in sparsely populated areas [28]. As such, the association between the likelihood of reporting chronic illnesses and subjective wellbeing as depicted in Hypothesis 2 may be strengthened by better health service availability, as stated in our third hypothesis:

**Hypothesis** **3** **(H3).** *The relationship between better subjective wellbeing and a lower likelihood of reporting chronic illnesses (Hypothesis 2) may be strengthened by better health service availability.*

## 3. Data and Methods

### 3.1. Data and Study Population

This study drew on three datasets. The HILDA survey data were provided by the Melbourne Institute, Australia [29]. This is a large-scale national longitudinal survey in Australia that was started in 2001, consisting of 7682 households and 19,914 individuals. Interviews were conducted with all adult members of each household annually to collect information at both the household and individual levels, including the presence or absence of a chronic illness, subjective wellbeing, their area of residence, and their demographic and socioeconomic characteristics. The HILDA survey data in different years (i.e., the survey conducted in 2001 was called wave 1) contained the consecutive participants that have been involved since the first wave and new participants in the latest year. Our study population was drawn from wave 16 of the HILDA survey (completed in 2016), consisting of 6662 individuals aged 50 or over (50–99) at the date of the interview. We defined the age threshold at 50 based on Yang et al. [30], who stated that people after age 50 are generally at a higher risk of chronic disease onset, and this age threshold is also consistent with the definition of older adults in other studies [3,31].

The second dataset was the number of general practitioners (GPs) and their service locations in 2016 collected from the Medical Directory of Australia (MDA)’s online data portal [32]. This dataset was geocoded to their postal address, and subsequently aggregated to the postal areas as defined by ABS [33]. The postal area unit is widely used in postal systems with names identifiable and unique across the whole of Australia; it also matches with the residential information (the area of residence) of the survey participants in the HILDA survey data. A total of 1099 postal units with the number of GPs were then assigned to the HILDA data at the individual level based on the post code.

The third dataset was the income and socioeconomic status data retrieved from the 2016 Australian Census of Population and Housing [34] via the online portal TableBuilder at the spatial unit of postal areas. The data included the total population, population aged 50 and above, and the Index of Relative Socioeconomic Disadvantage (IRSD) to reflect the socioeconomic level of the area where the respondent resides. These area-based data were then assigned to the HILDA data at the individual level based on the post code.

### 3.2. Measurements

#### 3.2.1. Chronic Illnesses

The HILDA data included information on whether respondents reported a chronic condition. These data were collected through face-to-face interviews with the participant. Participants were asked a yes/no question, such as “Do you have any long-term health conditions, impairment, or disability that restricts you in your everyday activities, and has lasted or is likely to last for six months or more, and cannot be corrected by medication or medical aids?” Respondents were then presented with a show card containing a list of 15 chronic health conditions as prompts to their response; however, their responses were not limited to these conditions. Thus, the likelihood of reporting a chronic illness is defined by a binary variable in the HILDA data indicating the self-reported chronic illness as yes (meaning they have at least one of the 15 chronic conditions) or no.

#### 3.2.2. Subjective Wellbeing

Subjective wellbeing is conceptually associated with populations with high cognitive and physical functional capacity [20]. As such, we used two measures of subjective wellbeing from the HILDA survey—general health and mental health—created from items in section A of the self-completion questionnaire. The measures of general health and mental health were drawn from the 2016 HILDA dataset. The level of general health was assessed based on the response to the following four questions, which asked how often in the past four weeks the respondent recognised himself as: (1) “I get sick a little easier than other people”; (2) “as healthy as anybody I know”; (3) “expect my health to get worse”; and (4) “my health is excellent”. The level of mental health was assessed by the response to the following nine questions, which asked how often in the past four weeks the respondent had: (1) “felt full of life”; (2) “been a nervous person”; (3) “felt so down in the dumps that nothing could cheer you up”; (4) “felt calm and peaceful”; (5) “have a lot of energy”; (6) “felt down”; (7) “felt worn out”; (8) “been a happy person”; and (9) “felt tired”. Possible responses were: (1) “all of the time”; (2) “most of the time”; (3) “a good bit of the time”; (4) “some of the time”; (5) “a little of the time”; and (6) “none of the time”. A person-specific raw score for each question was estimated as 1 to 6 based on possible responses, and then the average was calculated and applied to missing responses. The final score of the nine questions was summed to a range from 0 to 54, which was then further scaled linearly to range from 0 (worst possible outcome) to 10 (best possible outcome). Details on the survey questions and measurements are explained in the HILDA survey manual [29].

#### 3.2.3. Health Service Availability

There are different types of healthcare providers in Australia, including GPs, medical specialists, allied health workers, and nurses. GPs at local clinics or community health centres are typically the first point of contact for medical service [35]. Thus, the number of GPs in an area is a good indicator of the local health service capacity. To control for the variation in the total population in each postal area, we used the number of general practitioners (GP) normalised by the total population in that postal area (per 10,000 people) as an indicator of health service availability. These data were then classified into three categories: poor (no GPs in the area), good (the number of GPs ranged from 1 to 20), and excellent (the number of GPs ranged from 21 up to 67), given that such a classification introduces clear differentiations across three categories, as well as an even distribution of the total post code areas in each category.

#### 3.2.4. Individual Characteristics

The individual characteristics included the demographic and socioeconomic characteristics of individuals retrieved from the 2016 HILDA dataset. These included age, gender, highest education level, household income, labour force status, and marital status. The measure of each characteristic is documented in Table 1.

### 3.3. Modelling Method

We commenced with a statistic description of all variables in two groups: the older adult group reporting chronic illness and those not reporting chronic illness. We then employed a binominal logistic regression (BLR) to examine how the likelihood of reporting chronic illness was associated with subjective wellbeing and health service availably, given that the dependent variable—the reporting of chronic illnesses or not—was a binary variable [36]. In this model, the independent variables included subjective wellbeing measures of self-assessed mental and physical health, as well as health service availability measured by the number of GPs per 10,000 people (Table 1). We also controlled for a set of covariates known to be correlated with both the incidence of chronic illness and subjective wellbeing, as were reported in previous studies [4,8]. These included age, gender, socioeconomic status, highest educational level, household income, labour force status, and marital status as the potential confounders between subjective wellbeing and chronic illnesses. The BLR model is written as:

(1) $$Log\frac{p}{\left(1-p\right)}=b_{0}+b_{1}\times X_{1}+b_{2}\times X_{2}+b_{3}\times X_{3}+b_{i}\times X_{i}$$

where p is the probability of self-reported chronic illness; $b_{0}$ is the constant; $b_{1}$ is the coefficient for self-assessed mental health ($X_{1}$); $b_{2}$ is the coefficient for self-assessed physical health ($X_{2}$*)*; $b_{3}$ is the coefficient for the GP indicator *(*$X_{3}$); and $b_{i}$ is a set of coefficients for the set of individual characteristics ($X_{i\;},i=1\;\mathrm{to}\;7)$.

We constructed four BLR models. Model 1 included the covariates to indicate the association between individual characteristics and the likelihood of reporting chronic illnesses. Model 2 included the measures of subjective wellbeing (self-assessed mental and physical health). Model 3 included both the covariates and the measures of subjective wellbeing (self-assessed mental and physical health), and Model 4 further added the measure of health service availability (the GP indicator). We ran the four models step-wise so that we could assess the effect of the covariates on the likelihood of reporting chronic illnesses and examine whether the addition of other variables would improve the model’s performance.

## 4. Results

### 4.1. Descriptive Summary of Variables

Among the total 6662 older adults aged at and above 50 (mean age = 64.4; SD = 10.2), there were 2832 (42.5%) older adults with chronic illnesses and 3830 (57.5%) without chronic illnesses. Table 2 summarises the mental and physical health, as well as the individual characteristics, across these two groups. The older adults with chronic illnesses who reported a higher level of mental health in level 8, 9, and 10 accounted for 20.7%, 19.1%, and 14.4% of the total, respectively; the corresponding percentages for the counterparts without chronic illnesses were 23.4%, 27.3%, and 27.7%, respectively. For physical health, the older adults with chronic illnesses were distributed more evenly in the levels 4–8 (as a medium level of physical health), while the counterparts without chronic illnesses tended to be in a better position regarding physical health (29% in level 8, 22.1% in level 9, and 13.6% in level 10). However, the distribution in the three types of GP index had no substantial differences between the older adults with and without chronic illnesses. Regarding individual characteristics, the mean age of older adults without chronic illnesses was 62.11, slightly younger than those with chronic illnesses (mean of 67.48), but their average annual household income was AUD 101,234, which is substantially higher than those with chronic illnesses (mean of AUD 67,896). Moreover, compared to the older adults reporting chronic illnesses, the counterparts without reporting chronic illnesses tended to have a larger proportion in employment and married status, a better socioeconomic status (level 7 to 10), and a higher educational level (level 5 to 8).

### 4.2. Modelling Outcomes and Hypothesis Testing

The results from Model 1 (Table 3) showed that, compared with older adults reporting chronic illnesses, the older adults not reporting chronic illnesses tended to be younger (odds ratio = 0.98, *p* < 0.01), with higher yearly household income (odds ratio = 1.249, *p* < 0.01), a higher education level (odds ratio = 1.021, *p* < 0.05), and a higher IRSD level, reflecting a better socioeconomic status (odds ratio = 1.086, *p* < 0.01). Meanwhile, not being in labour force, being divorced, and having never married appeared to be significantly relevant to the likelihood of reporting a chronic illness. Model 1 had an R^2^ of 0.186; though not impressively high, this indicated that these individual characteristics partially explained the likelihood of reporting chronic illnesses. As such, our Hypothesis 1a, “old adults in better socioeconomic positions (e.g., higher income and well-educated) are less likely to report chronic illnesses”; Hypothesis 1b, “an increased age is associated with a higher likelihood to report chronic illnesses”; and Hypothesis 1c, “gender and marital status are not associated with chronic illnesses” can all be partially accepted. We observed across the four models that the association between these individual characteristics with the likelihood of reporting chronic illnesses remained relatively consistent, and the performance of the four models generally improved with the addition of subjective wellbeing (measured by mental and physical health) and health service availability variables, reflected by the increased R^2^ from 0.186 to 0.465 and the increase of the overall correct percentage of prediction from 67.3 to 78.1%. This indicated that subjective wellbeing and health service availability contributed to explaining the likelihood of reporting chronic illnesses.

Model 2 explored the relationship between chronic illnesses and subjective wellbeing. Compared to older adults with chronic illnesses, those without chronic illnesses also reported better physical health (odds ratio = 1.536, *p* < 0.01). As subjective wellbeing measures may capture both mental and physical health, this suggested that older adults without chronic illnesses had better subjective wellbeing. With the addition of individual characteristics, Model 3 showed that older adults not reporting chronic illnesses were in a slightly better mental health condition (odds ratio = 1.034, increased from 1.011 in Model 2, *p* < 0.1) and much better physical health condition (odds ratio = 1.779, increased from 1.536 in Model 2, *p* < 0.01). This indicated that the relationship between chronic illnesses and subjective wellbeing was strengthened by the addition of individual characteristics, including age (odds ratio = 0.967, *p* < 0.01), household income (odds ratio = 1.184, *p* < 0.01), IRSD (odds ratio = 1.063, *p* < 0.01), and not being in the labour force (odds ratio = 0.528, *p* < 0.01). In other words, the relationship between chronic illnesses and subjective wellbeing was strengthened for old adults with a relatively younger age, higher household income, better socioeconomic status, and being in the labour force. As such, our Hypothesis 2, “controlled by individual characteristics, older adults with better subjective wellbeing are less likely to report chronic illnesses”, can be accepted.

Finally, Model 4 showed that older adults without chronic illnesses had better subjective wellbeing (odds ratio = 1.034 and 1.781 for mental health and physical health, slightly increased from 1.034 and 1.779 in Model 3, *p* < 0.01). We further observed that good and excellent health service availability were negatively associated with reporting chronic illnesses (coefficient = −0.188 and −0.289, *p* < 0.1), and R^2^ increased from 0.445 in Model 3 to 0.465 in Model 4, indicating that those without reporting chronic illnesses were associated with better subjective wellbeing compared to older adults reporting chronic illnesses. Furthermore, this relationship was strengthened for older adults residing in areas with less health service availability (odds ratio = 0.829 for a good GP level and 0.749 for an excellent level, *p* < 0.1). As such, our Hypothesis 3, “the relationship between better subjective wellbeing and a lower likelihood of reporting chronic illnesses (Hypothesis 2) may be strengthened by better health service availability”, can be rejected. In other words, the availability of health services might not be attributable to the likelihood of reporting chronic illnesses, possibly due to the nature of chronic illnesses that might be more related to the lifestyle, living condition, and psychological and physical condition of human beings over a longer term [37]. Furthermore, particularly in the Australian context, healthy older adults may tend to reside in suburban areas closer to the natural environment and away from the inner-city areas with more noise, traffic, air pollution, and other urban environmental problems, although the health service availability is better in inner cities than the outer suburbs.

## 5. Discussion

### 5.1. Main Findings

Drawing on a sample of the older adults in Australia aged at or above 50 as the study population, this study examined the relationships among chronic illnesses, subjective wellbeing, and health services availability, and how such relationships are controlled by the individual characteristics of older adults. Drawing on data from three sources―the geo-coded data of general practitioners from MDA’s online data portal, the survey data from wave 16 of the HILDA dataset, and the census data of population and housing from ABS―we investigated whether older adults with better subjective wellbeing and living in areas with better health service availability are less likely to have chronic illnesses by holding their individual characteristics as covariates and testing three hypotheses drawn from the empirical findings in the existing scholarship. Our findings show that the relationship between the likelihood of reporting chronic illnesses and subjective wellbeing is varied by the older adults’ socioeconomic positions. Specifically, older adults with a higher educational level and higher household income, and with a stronger sense of subjective wellbeing, have a lower likelihood of reporting chronic illnesses, although health service availability does not appear to contribute to the relationship between subjective wellbeing and chronic illnesses.

### 5.2. Contributions to the Literature

The association between better socioeconomic status and the lower likelihood of reporting chronic illnesses in the Australian context is in line with the empirical observations found in a large body of studies in European countries (e.g., UK, Finland, Spain, Poland, Germany) and African countries (e.g., South Africa, Uganda) [11,15]. More specifically, our findings are consistent with the most observed relationship between lower income groups and poorer self-rated health [12,13,17,19], and align with the weakening association between socioeconomic positions and subjective health when individuals age [3]. What is less clear is how the likelihood of reporting chronic illnesses is associated with gender, educational level, and marital status. It runs counter to the previous observations that gender significantly affected the status of chronic illnesses [11]. Thus, the relationship between individual characteristics and chronic illnesses awaits further investigation through more robust empirical studies and the involvement of other potential confounder factors.

By holding the individual characteristics as covariates, our analysis suggests the association between a lower likelihood of reporting chronic illnesses and a stronger self-assessment of subjective wellbeing as a combinatorial measure of self-assessed mental and physical health. Although such an association has been commonly observed in previous studies [21], it is arguably more arbitrary if chronic illnesses are broken down to different forms [3], or if wellbeing is defined and measured in different ways [23]. Although the common explanation is that a stronger sense of wellbeing may serve as a solid base of mental resilience to protect against the onset of chronic illnesses [22], it is less clear about the mechanism of how subjective wellbeing affects the likelihood of reporting chronic illnesses without involving clinical experiments and diagnosed measures. Moreover, the involvement of individual characteristics strengthens the association between subjective wellbeing and chronic illnesses, which perhaps reflect the impact of individual characteristics as unobserved and observed moderators at baseline on wellbeing. However, such an association is subject to different types of chronic illnesses, and the specificity of such an association has not been widely investigated or confirmed in previous studies [38]. Furthermore, in contrast with the results shown in other studies [36,37,38] that the association between wellbeing and chronic illnesses tends to be stronger at younger ages, we do not observe a significant age dependency in our study. The reason of these divergent findings is unclear. Thus, further research to explore the causality, especially the causal direction between subjective wellbeing and chronic illnesses, is needed to confirm our findings.

The older adults living in an area with better health services availability are unexpectedly associated with a higher likelihood of reporting chronic illnesses in our study, which runs counter to the hypothesised expectation that the better availability of health services in local suburbs may lower the likelihood of reporting chronic illnesses [27]. This finding is possibly explained by the nature of chronic illnesses, which is combinatorically exogenous and endogenous, as the accumulative consequence of lifestyle, diet and nutrition, daily activities and routines, personal resilience to difficulties in life, and physical and psychological health [36]. Easy access to local health services may help to diagnose and treat chronic illnesses more effectively, however, it would be less persuasive to attribute the better availability of health services to the lower likelihood of reporting chronic illnesses without knowing how much or how often people actually use such health services. In addition, our findings may be specific to the Australian context that is highly suburbanised and car-dependent, where people can have relatively easy access to healthcare through driving. Australian urban planning and health planning advocates the equal configuration and access to health facilities to maximum social justice and equality [39]. Although large-scale public hospitals are more concentrated in inner cities, the distributions of GPs, dentists, private clinics, pharmaceutical services, and nursing homes are dispersed across suburban areas as a part of the planning regulation. Australians may enjoy the outdoors, recreation, and leisure, and this unique lifestyle makes Australian families tend to live in suburbs with more living space and proximity to the natural environment, where there may not be substantial differences in health service availability [39]. Early studies suggest that in some developing countries (e.g., South Africa), older adults may experience difficulties in managing their chronic conditions if there were not health services nearby their home, because they were heavily reliant on public health facilities for their treatment and have to access healthcare through public transportation or walking [11]. Finally, it is also worth noting that our measure of health services availability only accounts for the number of GPs; the finding may be different if the measure is extended to other kinds of health services (e.g., large public hospitals and private clinics).

Our analytical outcomes yield several policy implications. First, the national health system (e.g., Medicare in Australia) needs to be strengthened to meet the growing challenge of chronic illnesses among the aging population. A future agenda for strengthening the health systems can arise from the urgent need to scale up and sustain the priority interventions, particularly for the socioeconomically disadvantaged older adults who have reported a higher likelihood of suffering from chronic illnesses. Second, it is recommended for public health policymakers to put more weight on improving mental health and subjective wellbeing of older adults, which may protect against the incidence of chronic illnesses in the long-term. Accordingly, health planning should increase access and configuration of mental health centres, supported by community-based mental health education and promotion programmes. Third, the national health system should contribute to chronic illness interventions by delivering a comprehensive range of health services to older adults with different demographic and socioeconomic status. For example, a higher waiver of medical cost and more frequent home care should be given to lower income older adults with less mobility; the service of nursing homes can be prioritised to the older adults above a certain age with severe chronic illnesses.

### 5.3. Limitations

There are several limitations in this study that future studies can draw on to extend our findings. First, the measure of subjective wellbeing combines self-assessed mental and physical health, which can be extended to multiple indicators, reflecting both the exogenous and endogenous nature of an individual’s wellbeing, including self-realisation, life satisfaction, level of happiness and pleasure, good relationships with family and friends, and experiences of positive life events. Second, our analysis did not differentiate the types of chronic illnesses. As evidenced by Okely and Gale [3], the relationship between better subjective wellbeing and lower incidences of chronic illness only applies to stroke, diabetes, and myocardial infarction, but not to arthritis and chronic lung disease. Therefore, future studies could explore how this relationship between wellbeing and chronic illnesses can vary by the specific type of illnesses. In addition, the subjective wellbeing is measured as self-assessed mental and physical health, which may be overwrapped with the measure of self-reported chronic illnesses, since the incidence of chronic illnesses is also subject to a wide range of physical and mental conditions. There are measurable and unmeasurable confounders existing in the dynamic relationship between mental and physical conditions, subjective wellbeing, and chronic illnesses, which need to be explored by more robust models (e.g., causality analysis) in the future. Third, the likelihood of reporting chronic illnesses was assessed using a self-reported measure, however, the validation of self-reported measures varies according to the chronic illness outcomes. Although some studies have reported a high agreement between self-reported and clinically derived diagnosis in some forms of chronic illnesses (e.g., cardiovascular illnesses, diabetes) [40,41], future studies are needed to better address the potential bias existing in this validation. Fourth, a number of potentially relevant covariates relating to older adults’ individual characteristics, including diet, nutrition, sleep quality, perceived stress, and body weight, were not included in our analysis; this could also be explored in future studies if such data are available. Fifth, health service availability can be measured more accurately and at finer scale than by postal area, and can also consider multiple types of healthcare facilities, including hospitals, medical clinics, mental health centres, and community health centres. Another direction is to involve the actual usage of healthcare facilities (e.g., hospital admissions) or to measure the proximity to healthcare facilities to reflect healthcare accessibility more realistically [42]; this may require additional data of road network connection and the location of healthcare facilities. Sixth, a neighbourhood environment or the environment of local communities, which have been thought to have a long-term effect on the formation of chronic illnesses, should be considered to extend the analytical dimensions, including green coverage, walkability, road network, and sport and recreational facilities [43]. Finally, our analysis was unable to tease out the causal direction between chronic illness and subjective wellbeing, as the data used in our study were captured in the same longitudinal survey. Future studies across different longitudinal surveys over a certain period of time have the potential to reveal the causality temporarily.

## 6. Conclusions

To conclude, this study provides empirical evidence for the complex relationship among chronic illnesses, subjective wellbeing, health service availability, and the individual characteristics of the older adult population. In addition to corroborating previous results regarding the association between wellbeing and chronic illnesses, the study extends the research scope to incorporate health service availability, which has not been largely discussed in previous studies. Based on our empirical findings, a number of policy implications have been raised to reduce the incidence of chronic illnesses, provide further insight regarding the mechanisms underlying the association between wellbeing and chronic illnesses, and construct health initiatives for successful aging, long life expectancy, and wellbeing in later life. The HILDA data are a valuable source for this longitudinal study. By involving multiple waves of the HILDA data and other sources of survey and census data, we call for cross-sectional, longitudinal, and inter-generational studies in the future to enhance our holistic understanding of chronic illnesses, subjective wellbeing, and health service outcomes.

## Figures and Tables

**Table 1:** Variables and covariates used in the binomial logistic regression model.

Dependent Variables	Definition
Self-reported chronic illness	0: No 1: Yes
**Independent Variables**	
Health service availability	
GP index	0: Poor; 1: Good; 2: Excellent
Subjective wellbeing	
Self-assessed mental health ^A^	0 to 10
Self-assessed general health ^A^	0 to 10
**Covariates**	
Individual characteristics	
Age *	50 to 99
Gender	0: Male; 1: Female
IRSD ^A^	1 to 10 (as decile)
Household income *^,B^	0 to 1,370,818 (AUD)
Highest education level	1: Undetermined; 2: Year 11 and below; 3: Year 12; 4: Cert III or IV; 5: Advanced diploma; 6: Bachelor or honours; 7: Graduate diploma/certificate; 8: Postgraduates—masters or doctorate
Current labour force status	0: Employed; 1: Unemployed; 2: Not in the labour force
Marital status	0: Legally married; 1: De Facto; 2: Separated; 3: Divorced; 4: Widowed; 5: Never married

Note: A: IRSD, the Index of Relative Socioeconomic Disadvantage, is a regional characteristic that has been assigned to each individual living in that area. We included the IRSD here because it reflects the socioeconomic level of the area where the older adults reside. The IRSD index is measured as a decile ranging from 1 to 10; an area with a higher IRSD index means that this area has a higher socioeconomic status. B: Household income is a numeric variable that was log-transformed from the raw data to ensure that the data are normally distributed. * These are numeric variables used in the models.

**Table 2:** Statistical summary of variables in older adult groups with/without reporting chronic illnesses.

Variable	Whether or Not Report Chronic Illnesses		Variable	Whether or Not Report Chronic Illnesses	
	Yes	No		Yes	No
Mental Health	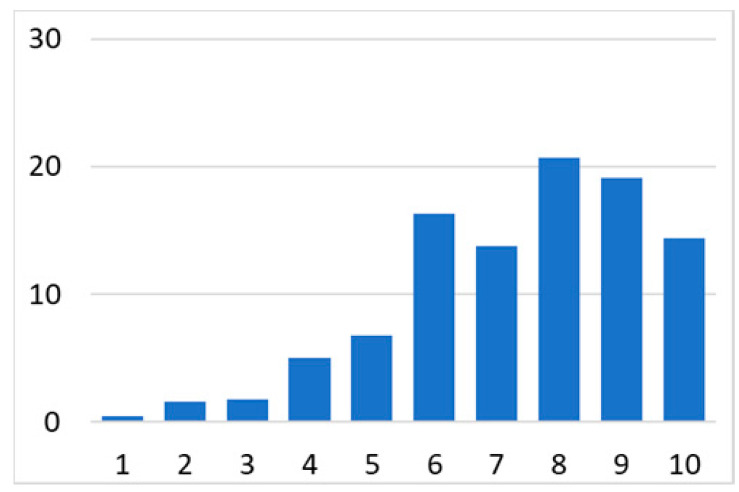	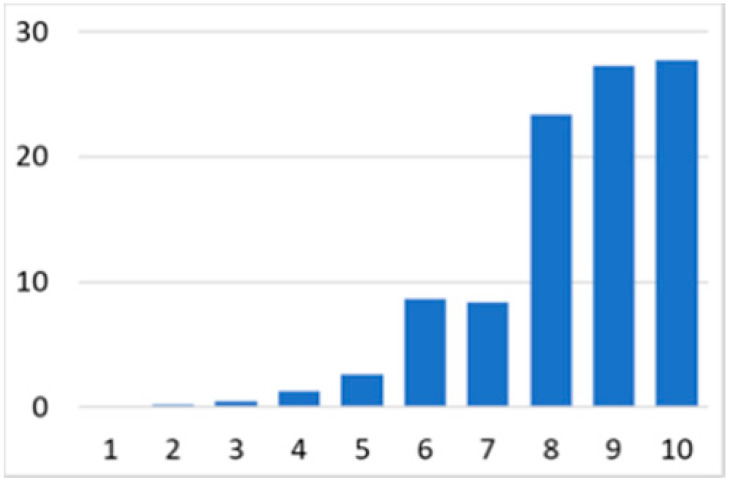	Physical Health	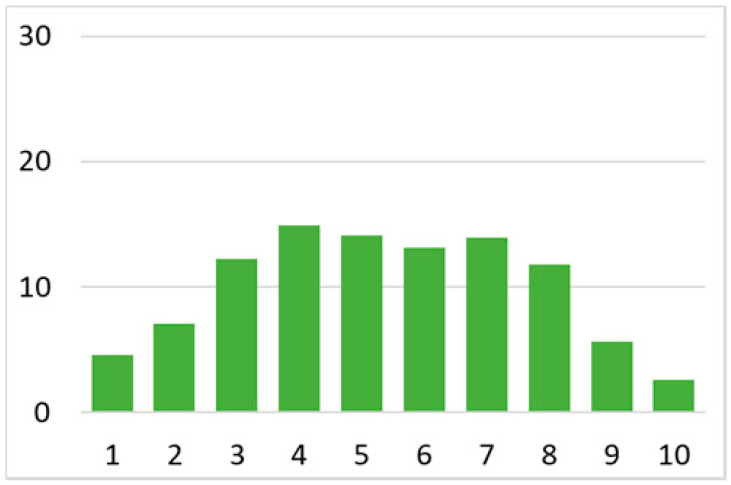	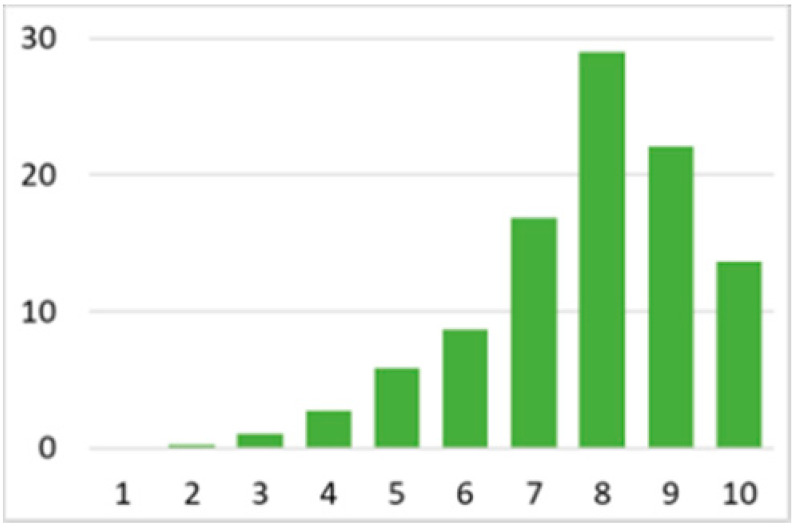
Labour force	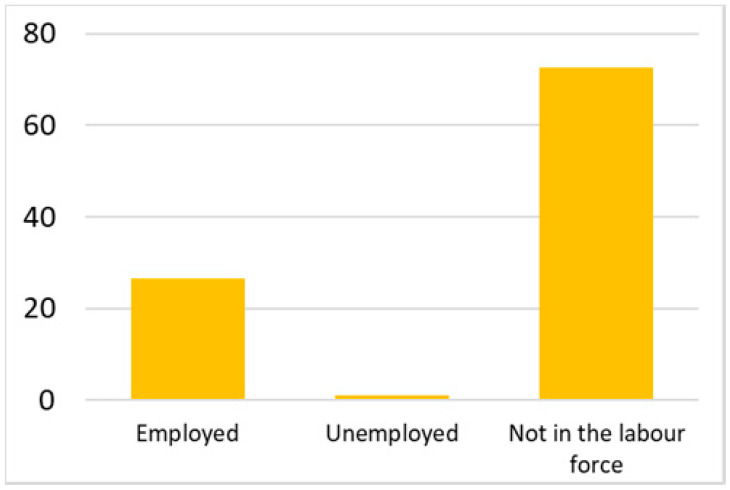	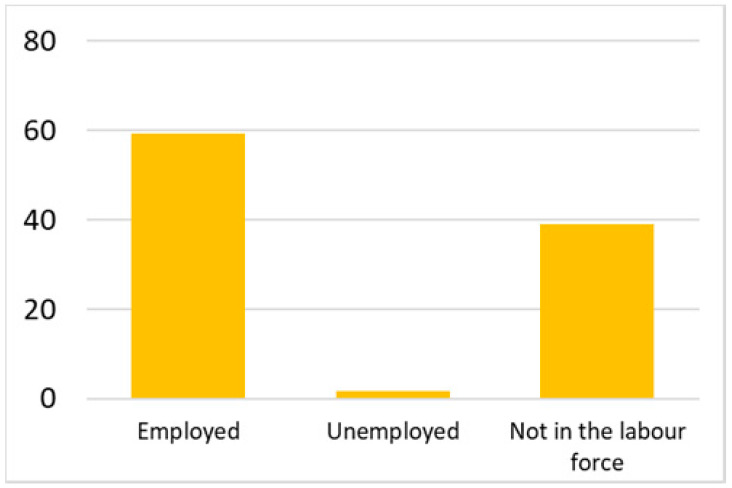	Marital status	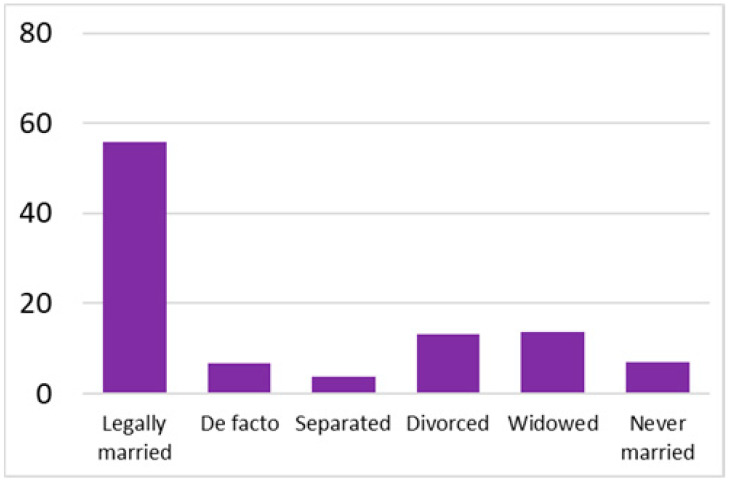	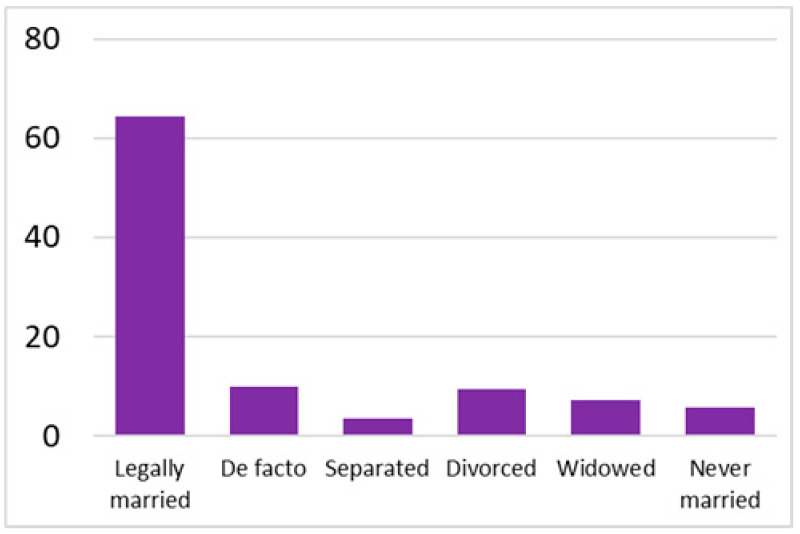
Highest edu level	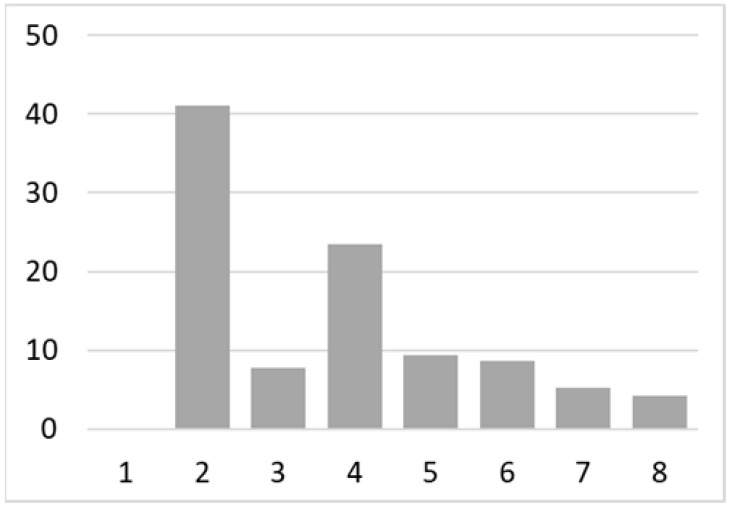	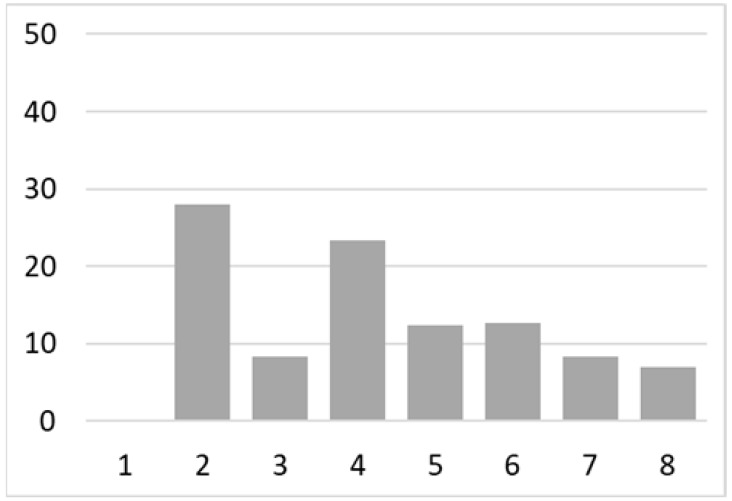	IRSD	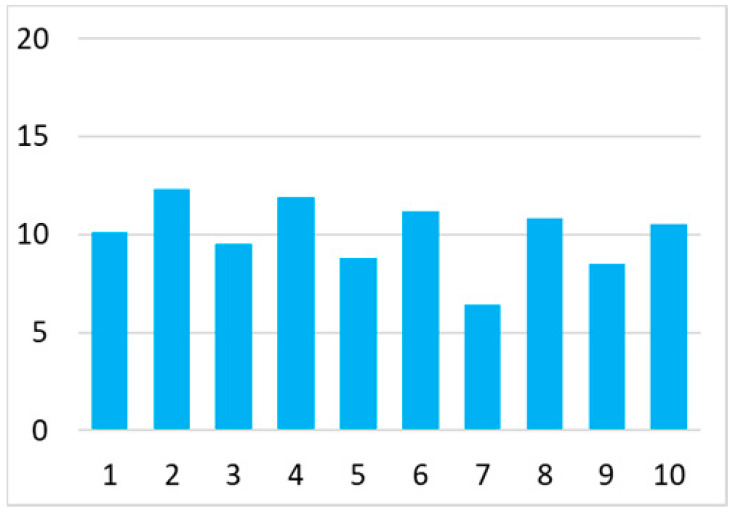	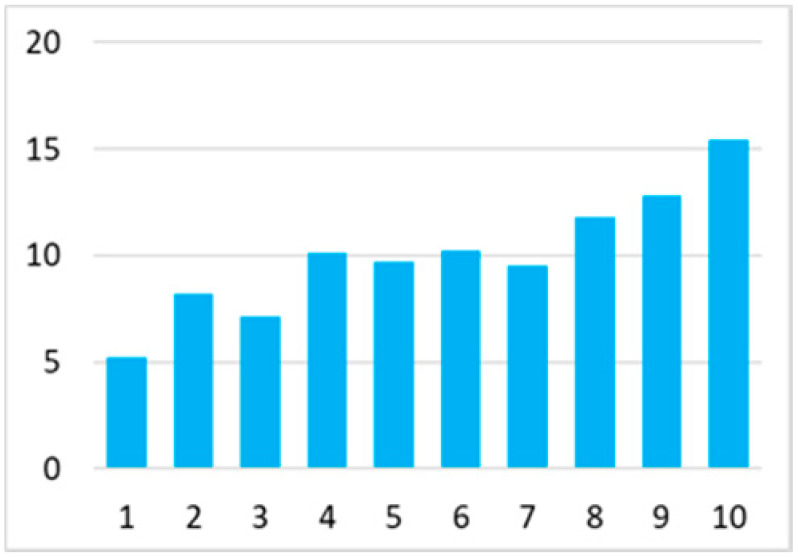
GP index	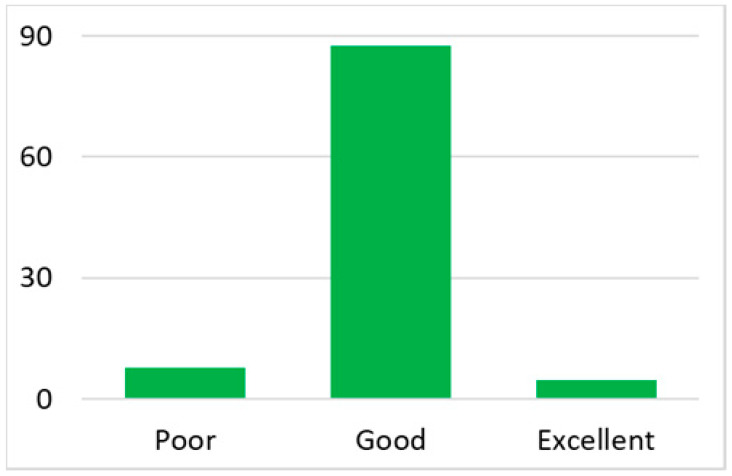	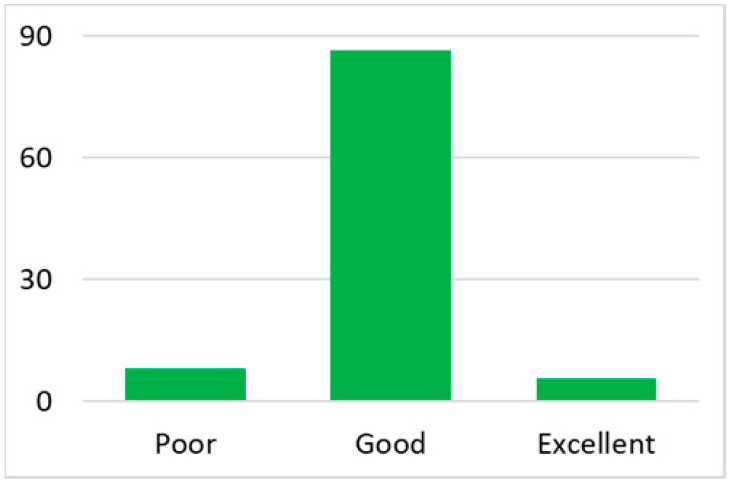	Gender	Male: 44.8% Female: 55.2%	Male: 48.0% Female: 52.0%
Age	Mean: 67.48 SD: 10.84 Range: 50–99	Mean: 62.11 SD: 9.09 Range: 50–99	HH Income	Mean: 67,895.75 SD: 57,367.31 Range: 0–993,088	Mean: 101,233.89 SD: 85,177.75 Range: 0–1,370,818

Note: The *y*-axis in the above figures is the percentage of the population in a certain category over the total population.

**Table 3:** Results of the binomial logistic regression.

	Model 1		Model 2		Model 3		Model 4	
	Coefficient	Odd ^A^	Coefficient	Odd ^A^	Coefficient	Odd ^A^	Coefficient	Odd ^A^
Constant	−0.794	0.452	3.782 ***	43.895	−3.469 ***	0.031	−3.357 ***	0.035
**Individual characteristics**								
Female	0.027	1.027 (0.920–1.146)			−0.113 *	0.893 (0.786–1.014)	−0.110 *	0.896 (0.789–1.017)
Age	−0.020 ***	0.980 (0.973–0.987)			−0.033 ***	0.967 (0.959–0.975)	−0.033 ***	0.967 (0.959–0.975)
Household income	0.222 ***	1.249 (1.146–1.361)			0.169 ***	1.184 (1.073–1.308)	0.173 ***	1.189 (1.076–1.313)
Highest educational level	0.021 **	1.021 (1.000–1.042)			0.003	1.003 (0.979–1.027)	0.003	1.003 (0.980–1.028)
Socioeconomic status (IRSD)	0.083 ***	1.086 (1.065–1.107)			0.061 ***	1.063 (1.039–1.087)	0.061 ***	1.063 (1.040–1.087)
Labour force (employed as ref)								
unemployed	−0.028	0.973 (0.614–1.540)			0.198	1.219 (0.719–2.067)	0.203	1.225 (0.722–2.080)
not in labour force	−1.000 ***	0.368 (0.321–0.422)			−0.638 ***	0.528 (0.450–0.620)	−0.637 ***	0.529 (0.451–0.621)
Marital status (married as ref)								
de Facto	−0.043	0.958 (0.785–1.169)			−0.025	0.975 (0.777–1.223)	−0.024	0.977 (0.778–1.225)
separated	−0.238 *	0.788 (0.592–1.049)			0.025	1.026 (0.722–1.456)	0.033	1.033 (0.728–1.467)
divorced	−0.371 ***	0.690 (0.577–0.826)			−0.261 **	0.771 (0.625–0.950)	−0.252 **	0.778 (0.630–0.959)
widowed	−0.003	0.997 (0.821–1.211)			0.085	1.089 (0.869–1.365)	0.094	1.098 (0.876–1.377)
never married	−0.385 ***	0.680 (0.542–0.854)			−0.166	0.847 (0.650–1.105)	−0.151	0.860 (0.659–1.122)
**Subject wellbeing**								
Mental health			0.011 *	1.011 (0.973–1.050)	0.033 *	1.034 (0.992–1.077)	0.033 *	1.034 (0.992–1.077)
Physical health			0.423 ***	1.536 (1.518–1.556)	0.576 ***	1.779 (1.714–1.846)	0.577 ***	1.781 (1.715–1.848)
**Health service availability** GP level (pool as ref)								
good							−0.188 *	0.829(0.663–1.036)
excellent							−0.289 *	0.749 (0.531–1.056)
R^2^	0.186		0.372		0.445		0.465	
Overall correct percentage of prediction	67.3		75.0		77.1		78.1	

Note: The reference group of the overall model is people with chronic illness (coded as 1). * *p* < 0.1; ** *p* < 0.05; *** *p* < 0.01; A: Numbers in the bracket underneath are the lower and upper boundary at a 95% confidence level.

## Data Availability

Wave 16 of the HILDA data can be requested through an application to the Australian Data Archive Dataverse at the Australian Government Department of Social Services (https://dataverse.ada.edu.au/) (accessed on 6 May 2021).
